# Analyzing pericytes under mild traumatic brain injury using 3D cultures and dielectric elastomer actuators

**DOI:** 10.3389/fnins.2022.994251

**Published:** 2022-11-10

**Authors:** Yi-Han Wu, Thomas I-H Park, Eryn Kwon, Sheryl Feng, Patrick Schweder, Mike Dragunow, Vickie Shim, Samuel Rosset

**Affiliations:** ^1^Auckland Bioengineering Institute, The University of Auckland, Auckland, New Zealand; ^2^Centre for Brain Research, The University of Auckland, Auckland, New Zealand; ^3^Department of Pharmacology, The Faculty of Medical and Health Sciences, The University of Auckland, Auckland, New Zealand; ^4^Auckland City Hospital, Auckland, New Zealand

**Keywords:** pericytes, mild traumatic brain injury, dielectric elastomer actuators, qRT-PCR analysis, 3D cell culture, stretch, strain, TBI

## Abstract

Traumatic brain injury (TBI) is defined as brain damage due to an external force that negatively impacts brain function. Up to 90% of all TBI are considered in the mild severity range (mTBI) but there is still no therapeutic solution available. Therefore, further understanding of the mTBI pathology is required. To assist with this understanding, we developed a cell injury device (CID) based on a dielectric elastomer actuator (DEA), which is capable of modeling mTBI via injuring cultured cells with mechanical stretching. Our injury model is the first to use patient-derived brain pericyte cells, which are ubiquitous cells in the brain involved in injury response. Pericytes were cultured in our CIDs and mechanically strained up to 40%, and by at least 20%, prior to gene expression analysis. Our injury model is a platform capable of culturing and stretching primary human brain pericytes. The heterogeneous response in gene expression changes in our result may suggest that the genes implicated in pathological changes after mTBI could be a patient-dependent response, but requires further validation. The results of this study demonstrate that our CID is a suitable tool for simulating mTBI as an *in vitro* stretch injury model, that is sensitive enough to induce responses from primary human brain pericytes due to mechanical impacts.

## Introduction

Traumatic brain injury (TBI) is defined as damage to the brain caused by an external mechanical force due to direct impact, rapid acceleration, object penetration, or explosion blast waves (Maas et al., 2008). These traumas consequently create changes in intracranial pressure and brain tissue deformation (Ghajar, 2000). The physical damage caused by TBI negatively affects brain function resulting in a temporary or permanent deficit in cognitive, physical, and behavioral activities (Parikh et al., 2007). TBI is the leading cause of death and disability for healthy young people under 45 years of age (Sariaslan et al., 2016). Worldwide the number of people who sustain TBI is estimated to be 69 million individuals (Dewan et al., 2019).

Currently, there are no therapeutic procedures or medications that can promote brain repair or reduce brain damage (Sharp and Jenkins, 2015; Kenzie et al., 2017), including those injuries in the mild severity range (mTBI), which make up to 90% of all TBIs. Successful *in vitro* models improve the understanding of the mechanisms for cellular impairment caused by TBI. Various experimental models have already been developed, including *in vivo* animal models, and *in vitro* models with cultured animal and human brain cells, where the majority of studies used animal cells (Arun et al., 2011; Febinger et al., 2015; Ravin et al., 2016; Sherman et al., 2016; Zanier et al., 2016). Unfortunately, translation of the outcomes from these experimental models have not been successful at the human clinical trial stages (Dragunow, 2020). Bridging this translation gap requires (1) the development of a testing system that better replicates key mechanism for tissue and cell injury from TBI (2) the utilization of more human brain cells to improve the accuracy of tested therapeutic methods in humans (Dragunow, 2008).

**FIGURE 1:**
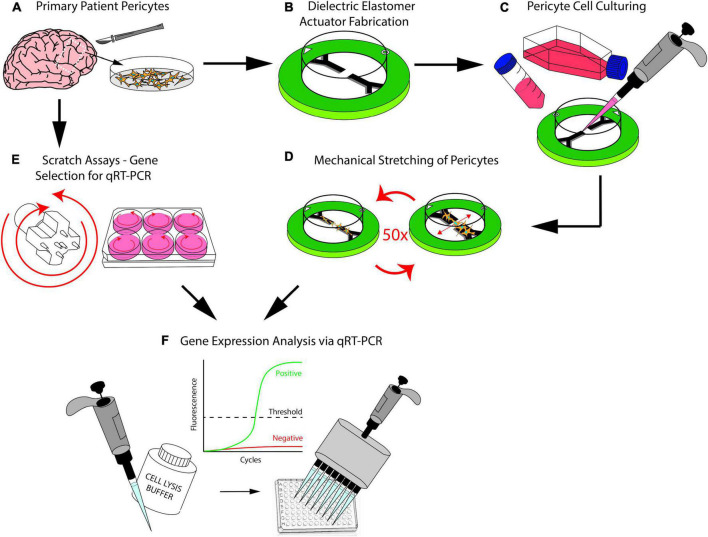
**(A)** Brain pericytes isolated from human explants. **(B)** Dielectric elastomer actuators that produce uniaxial strain are fabricated. **(C)** Patient-matched brain pericyte cells are plated on our devices. **(D)** Our devices stretch the cultured pericyte cells 50 times at 20% strain. **(E)** Scratch assays were conducted using a custom-3D-printed scratch device as a preliminary step to determine which genes would respond to injury. **(F)** Polymerase chain reaction technique used to determine the genes that responds to injury from the scratch assays, the same genes were then investigated after injuring the pericytes with our stretch devices.

**FIGURE 2:**
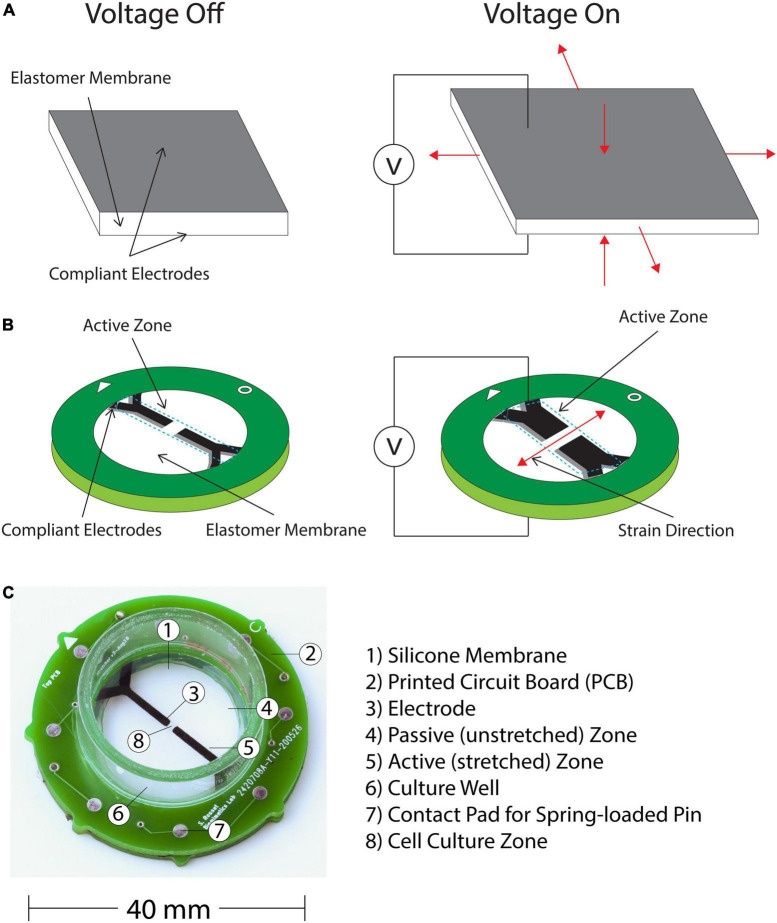
**(A)** DEAs consists of an elastomer membrane located between two compliant electrodes. When a voltage is supplied charges of opposite signs accumulate on the electrodes, which causes the membrane thickness to decrease and the surface area to increase. **(B)** Applying the operation principle of DEAs to mechanically insult cells with a uniaxial stress and high strain rate. This is achieved by applying an anisotropic prestrain to the membrane, which leads to a directional actuation strain. Our device consists of this prestressed membrane on a frame with two sets of compliant electrodes defining the actuation zone. The pericyte cells are cultured in the active zone, where they are stretched and injured when a voltage is applied. The center of the device that is not covered by the electrodes is transparent, which enables observation of the assay under a microscope. **(C)** Final Complete CID with individual components labeled.

In the present study, we address both of these requirements by demonstrating the viability of using dielectric elastomer actuators (DEAs) to recapitulate mTBI in an *in vitro* model with primary human brain cells (Figure 1). We report the findings from the characterization of DEAs as well as the mechanobiological changes in these patient-matched brain pericyte cells after mechanical stretch. The overall aim is to show that (1) human brain cells can be cultured on DEAs (Figure 1C); (2) these can be submitted to a controlled amount of strain (Figure 1D) that is large enough to provoke a biological response (Figure 1F). This will contribute to the long-term aspiration of reproducing a realistic and representative strain profile of human TBI *in vitro* to measure cellular responses after brain injuries.

Moreover, to satisfy the first requirement of the translation gap, we developed a cell injury device (CID) based on a DEA. DEAs are deformable capacitors that comprise a dielectric elastomer membrane located between two compliant electrodes. When a voltage is supplied to the pair of electrodes, charges of opposite signs accumulate on each electrode, which causes the membrane to decrease in thickness and expand in surface area (Pelrine et al., 2000; Figure 2A). DEAs have applications in the various fields of soft robotics, tuneable optics, and bioengineering. There have been previous reports of applying DEAs to stretch cells, such as the cyclic stretching of Caco-2 cells (Cei et al., 2016) and induction of cytoskeleton changes in fibroblasts via dynamic stretching, thus mimicking mechanical stimulation of tissues in a physiologically relevant manner (Costa et al., 2020). As well as strain-induced alignment of lymphatic endothelial cells (Poulin et al., 2018a) and rapid stretching of cardiac tissue (Imboden et al., 2019).

*In vitro* TBI models can be categorized into the five major methods of mechanical loading, which are blast, compression, scratch, shear, and stretch (Wu et al., 2021). The injury model presented in the current study falls under the stretch category, which is the most commonly used loading type in TBI studies because maximum principal strain resulting from tissue stretch is regarded as an effective predictor of TBI (Gabler et al., 2018; O’Keeffe et al., 2019). Our device applies tensile strains in plane directly to cell-seeded substrates as in other similar devices (Sturdivant et al., 2016; Nakadate et al., 2017; Bianchi et al., 2019; Li et al., 2019; Braun et al., 2020). However, the advantage of having the actuator embedded in the culture membrane is that applied loads are directly transferred to the cells or tissues being injured. This is in contrast to other common designs that utilize pneumatic cylinders to apply stretch, where the applied strains can only be estimated (Wu et al., 2021).

The operation principle of DEAs (Figure 2A) can be applied to mechanically load cells (Cei et al., 2016; Poulin et al., 2016, 2018a,b; Costa et al., 2020), which can lead to injury if the insult is of sufficiently large amplitude and/or applied with a high strain rate. DEAs are devices utilized in our study because they are compact and easy to use on a microscope, with great potential for future development of constructing arrays of these actuators for high-throughput assays. Additionally, DEAs are lightweight, have high strain capabilities (>10%), high electromechanical efficiency, and, most importantly for TBI applications, a fast response time, with strain rates up to 870 s^–1^ as demonstrated on a DEA-based cell-stretching device (Poulin et al., 2018a). For this study we aim to produce 20% strain or greater, which falls within the mild spectrum of TBI (mTBI) (Skotak et al., 2012).

Furthermore, to address the second need in the translation gap of using human brain cells, the cell type selected for this study is human brain pericytes. Pericytes are perivascular cells found ubiquitously throughout the central nervous system (Brown et al., 2019). In the brain, pericytes are located on the vascular endothelium (Sweeney et al., 2016). Pericytes play significant roles in the brain including the regulation of neuroinflammation, angiogenesis, and blood-brain barrier permeability (Armulik et al., 2010; Sweeney et al., 2016). Furthermore, some evidence suggests that post-injury, pericytes are heavily involved in scar formation (Goritz et al., 2011; Greenhalgh et al., 2013; Soderblom et al., 2013; Trost et al., 2016; Dias and Göritz, 2018). Despite these critical functions’ involvement in TBI, there has been a conspicuous gap in the literature that utilize pericytes in the *in vitro* models (Wu et al., 2021). As such, we chose to use patient-matched brain pericytes in our TBI model.

To the best of our knowledge, the findings presented in this study are the first *in vitro* injury model conducted using primary human brain pericyte cells. The results of this study could potentially lead to the discovery of a therapeutic target for mTBI, as well as increasing our understanding of the mechanisms of pericyte cells’ involvement after traumatic mechanical insults to the brain.

## Materials and methods

The expansion of a DEA is intrinsically equi-biaxial (Figure 2A), however, directionality of the mechanical stress can be controlled via design specifications. Since we aim to apply a uniaxial strain to our pericyte culture, we applied an anisotropic prestrain to the culture membrane, which leads to a directional actuation strain due to the non-linear hyperelastic properties of the silicone membrane (Akbari et al., 2013). Our CID is based on the work of Poulin et al. (2016) and illustrated on Figure 2B. It consists of a prestressed membrane on a frame with two sets of compliant electrodes defining the actuation zone. The cells can then be cultured in the active zone of the device, where they are stretched and injured according to the applied voltage. The center of the device, which is not covered by the electrode remains transparent, hence enabling observation of the assay under a microscope.

### Cell injury device fabrication procedure

The fabrication procedure of our CID is based on our previous work (Poulin et al., 2018b). We briefly describe this here with an emphasis on the adaptations and changes introduced for our study with human brain pericytes.

First, a Wacker Elastosil^®^ film 2030 silicone membrane is biaxially prestretched 2.7 and 1.2 times its original lengths along the two perpendicular directions (Supplementary Figure 1A). Due to the incompressibility of silicone, the thickness of the membrane decreases from 50 μm before the pre-stretch to less than 20 μm after. This highly anisotropic pre-stretch effectively increases the membrane stiffness along the high pre-stretch axis (Poulin et al., 2016), and hence, when voltage is applied, the device actuates primarily in the low prestretch direction, leading to a uniaxial actuation strain (Figure 2B). We prestretch a membrane from initial dimension 100 × 200 to 270 × 240 mm^2^ (Supplementary Figure 1A). The silicone chosen for this study was selected for its actuation properties. It combines a high dielectric breakdown field (80–100 V/μm), a large elongation at break (450%) and tear strength (10 N/mm). The membrane can therefore sustain the large prestretch and the electric field required for actuation. After prestretch, we glue 16 52 mm-diameter rings on the membrane using silicone adhesive, thus leading to 16 prestretched membranes (Supplementary Figure 1A). The rest of the process is applied to each of the 16 rings separately, to fabricate a batch of 16 actuators.

The compliant electrodes are formed by spray-coating the membrane with an electrically conductive ink through a shadow mask (Supplementary Figure 1B). The ink consists of a mixture of carbon black (Ketjenblack EC-300J), silicone (Silbione LSR 4305), and silicone solvent. This process is repeated for both sides of the membrane and the electrodes are then cured at 80°C for 30 min (Supplementary Figure 1B).

Printed circuit boards (PCB) are glued on either side of the membrane (Supplementary Figure 1C). They provide a structural frame that holds the membrane prestretch, as well as the electrical connection to the compliant electrodes on the membrane. The bottom side of the PCB, which is in contact with the membrane has large square metallic contact pads that align with the carbon electrodes coated on the membrane. Good electrical contact between the pads and electrodes is ensured by applying a thin layer of a conductive silicone paste (Silicone Solutions^®^ SS-24 Electrically Conductive Silicone RTV) on the PCB contact pads prior to gluing them to the membrane. The membrane between the outer diameter of the PCB and the initial frame is then cut with a scalpel, and the initial frame that was holding the prestretched membrane is discarded (Supplementary Figure 1D).

To contain the media required for cell culture, a section of polycarbonate tube (diameter 22 mm, height 10 mm) is glued to the top PCB and acts as a well for the cell culture. The finished CID is shown in Figure 2C.

Post fabrication, the strain vs. voltage behavior of each CID was characterized. This allowed us to calculate the voltage required for each CID to reach 20% strain. Since the electrode spray coating was done separately for individual CID, differences between each device inevitably occurred, requiring the characterization of each fabricated device. Additionally, this procedure provides a quality check to ensure the devices are functioning as expected.

### Design parameter optimization

For in-plane DEAs that consists of a prestretched membrane separated into an active zone (with electrodes), and a passive zone (without electrodes), the mechanical tension of the passive zone absorbs the expansion of the active zone. It has been shown that the actuation performance is linked to the relative size of the passive zone, with the optimum being when the uncoated zone is infinitely larger than the active area (Koh et al., 2011; Rosset et al., 2015), which is not practical. Using an analytical model of a circular actuator at the center of a circular membrane, Koh et al. (2011) have shown that the impact of the passive area becomes negligible, if its diameter is at least 10 times larger than that of the active area.

For our device, there was a trade-off between having a large actuation zone to stretch large number of cells for subsequent molecular experiments, and being able to reach 20% strain before dielectric breakdown. Consequently, we tested for a passive membrane with a fixed size of 22 mm, with three different widths of electrodes (i.e., the active zone): 6, 3, and 1 mm to investigate the influence of the stretching zone size on the actuation strain, and select an optimal design.

Cell culture work requires a high level of sterility to prevent contamination (Tipnis and Burgess, 2018). Therefore, we analyzed the impact of different sterilization methods on our CIDs. Our analysis investigated three aspects (1) can the device survive the sterilization process and (2) is the actuation impacted by the sterilization process? (3) what is the impact of incubation post cell plating which can last up to 72 h at 37°C in saturated humidity?

The three sterilization methods that we investigated are ultraviolet (UV) light/Ozone treatment (via the UV/Ozone ProCleaner), isopropanol (IPA), and autoclaving, all methods that could reach levels of disinfection acceptable for medical purposes (Dempsey and Thirucote, 1989). The UV/Ozone treatment was the preferred method of choice given it was previously used for the sterilization of polydimethylsiloxane stamps for other work conducted in our lab. The effects of this treatment were tested by subjecting the CIDs to the UV/Ozone for 30 min inside the UV/Ozone ProCleaner. The autoclave process involves subjecting the CIDs to 121°C for 15 min in a closed chamber. While the IPA treatment includes full submersion in IPA for 1 h before being dried in a controlled environment.

The strain capabilities of 16 CIDs pre- and post-sterilization were measured and characterized for each of the three aforementioned methods. These 16 CIDs all had the same electrode width (1 mm) and strains in each electrode under different sterilization methods were measured. The CIDs were characterized in air pre-sterilization, without any liquid present in the media well, as a reference point to measure the effects of the different sterilization methods. After the post-sterilization characterizations, the CIDs were incubated for 72 h at 37°C and characterized once again. This Incubation had no significant impact on the actuation strain.

The strain of the actuator as a function of applied voltage is measured using an optical camera and a Peta-pico-Voltron high voltage power supply (Schlatter et al., 2018) controlled with a LabVIEW program. The corners of the electrodes are tracked to measure the global strain of the active area.

### Ethics and human tissue processing

The experiments conducted here were approved by the Northern Regional Ethics Committee (New Zealand) and The University of Auckland Human Participants Ethics Committee (Approval number: AKL/88/025/AM2217). All methods were carried out in accordance with the approved guidelines. Biopsy human brain tissue was obtained with informed written consent from the patient and family members. Tissues used in this study were derived from neurosurgical procedures for intractable epilepsy from patients that were drug resistant. Access to these biopsy tissues is only possible because of the collaboration with Auckland City Hospital. Where the donated cells are cultured for a few months and passaged at least five times prior to being implemented in any experimental work. Earlier work conducted in our lab (Jansson et al., 2014; Smyth et al., 2018a) has demonstrated that this processing reduces the effects of proinflammatory cytokines often exhibited in *in vitro* pericyte cell cultures (Brown et al., 2019; Yamanaka et al., 2021). Thereby, simulating experiments as close to healthy primary human pericyte cells as possible.

### Cell culture protocol

Patient-derived epilepsy brain pericytes are isolated from processing brain tissue post-epilepsy surgery. This isolation procedure was previously described in greater detail in our previous work (Park et al., 2022). The characterization of the pericyte cell lines used in this study were also described previously in our earlier studies (Park et al., 2016; Smyth et al., 2018b). Cells are incubated at 37°C with 5% CO_2_ and grown in flasks until seeding for the experiment, usually 4–5 days. The purity and distribution on the CIDs of the primary human pericytes used in this study was verified by immunostaining for pericyte markers platelet derived growth factor receptor-β (PDGFRβ) and α-smooth muscle actin (αSMA) (Supplementary Figures 2, 3). These results authenticate the purity of our experimental pericytes as there were Hoechst positive nuclei associated with PDGFRβ positive staining (Supplementary Figure 2). Concurrently with the cell growing, the CIDs are sterilized by autoclaving in preparation for cell culturing. The autoclaving cycle uses pressurized steam to hold the temperature at 121°C for 15 min. Post autoclaving, the DEAs are incubated at 65°C until they are dried.

Once desired confluency of the pericytes is reached, flasks are trypsinised with 0.25% trypsin-ethylenediaminetetraacetic acid (EDTA) that dislodges the pericytes from the bottom of the flasks. Cells are counted with a hemocytometer, and the media volume is adjusted to get a desired cell density of 1,500,000 cells/mL. Next, this cell suspension is combined with an equal volume of 1:2 diluted Matrigel, which results in a final cell density of 750,000 cells/mL.

It is well known that cellular adhesion and migration are related to the rigidity of the culture substrate (Vertelov et al., 2016). However, for our study, the silicone membrane was chosen for its actuation properties. We conducted cytotoxicity tests to verify the compatibility of the substrate with pericytes. While cell death is not an issue, obtaining reliable cell adhesion on the silicone membrane is delicate. To solve this issue, the cell plating on the CIDs is done as a mixture in conjunction with Matrigel, a gelatinous protein mixture resembling the complex extracellular environment in cell culture. The active zone of the CIDs is targeted with the cell suspension and Matrigel mixture as this is where the strain is generated. The Matrigel is made from thawing a frozen Matrigel aliquot and diluting it 1:2 with Dulbecco’s Modified Eagle Medium/Nutrient Mixture F-12 (DMEM/F12) cell culture medium (Gibco^®^). This high viscous Matrigel is then combined with an equal volume of pericyte cell suspension, as previously mentioned, which results in a final Matrigel dilution of 1:4. The pericyte cell suspension is prepared separately in conjunction with the Matrigel solution before being mixed for cell plating.

After obtaining the final cell density and Matrigel dilution, 35 μL of this mixture is plated on each CID along the active zone (Figure 2B), which results in a total of 26,250 cells per CID. This targeted plating ensures that most of the plated cells are submitted to mechanical stretch. As a result of the highly viscous Matrigel-cell suspension, this targeted plating will also create a 3D cell culture rather than a monolayer 2D cell culture.

Following plating, the CIDs are incubated for 2 h before the pericytes are checked under the microscope for adequate adherence. After sufficient adherence, the CID well is then filled with 1 mL of fresh media to provide the cells with the necessary nutritional sustenance for growth and survival. We used the blend of DMEM/F-12 liquid medium with 10% Fetal Bovine Serum (FBS) and 1% Penicillin Streptomycin Glutamine (PSG). After adding media, the CIDs are incubated for 3–4 days until the cells reach confluency, changing 50% media every 1–2 days, in preparation for mechanical stretching.

### Scratch assay protocol

Due to the lack previous work on mechanobiological response of human brain pericytes after TBI, there was a need to screen for genes of interest involved in injury response. For this purpose, we chose to utilize a well-established cell injury model, scratch assays. The pericytes were prepared in the same manner as described above. After obtaining the desired density, a 6-well plate had 3 mL of cell suspension plated in each well, which results in a total of 150,000 cells per well. This 6-well plate is used for two time points, 4 and 48 h. Following plating, the pericytes are incubated at 37°C with 5% CO_2_ for 3–4 days till full confluency is reached. Then, the pericyte monolayer is injured with multiple circular lacerations with an autoclaved custom-made scratch device. This scratch device comprises of a handle with six pins that fit snuggly into a well on a 6-well plate, and is designed to rotate such that the six pins inflicts six circular lacerations on the pericyte monolayer (Figure 1E and Supplementary Figure 4). Post-injury, the cells are lysed after 4 and 48 h, with the control and scratched wells collected into separate Eppendorf^®^ tubes. These tubes are then stored at −80°C before RNA purification and examination of gene expressions via quantitative real-time polymerase chain reaction (qRT-PCR) analysis. The protocols for both techniques are described in the qRT-PCR protocol section.

### Protocol for stretching assay on pericytes with cell injury devices

**FIGURE 3:**
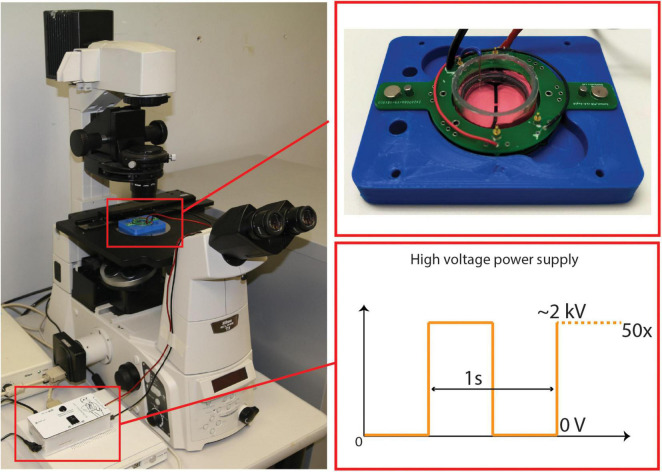
CID Stretching Apparatus Setup—includes a microscope connected a live camera feed, and a power supply attached to the CID. A close up of a cultured CID placed in the holder and a representation of the square signals used to apply strain.

Once the desired confluency was achieved, the cells were subjected to mechanical insult. The cultured CID is placed in a holder that maintains the device in place under the microscope, while providing electrical connections to the PCB. The holder is attached to a Peta-pico-Voltron high voltage power supply generating a square output waveform (Schlatter et al., 2018). The use of a square waveform leads to the highest achievable strain rate in open-loop. A live camera records the stretching of the pericytes and captures static images to confirm strain values post-injury (Figure 3). Based on a strain-voltage characterization of each device made after fabrication, the required voltage to apply to the CID to obtain a strain of 20% can be calculated. The following settings were used: 50 cycles at 20% strain, applied at 1 Hz. Local strain mapping of the cell culture under stretch has been performed in the transparent part of the active area using a subpixel phase-based image registration algorithm performed on images acquired during the stretching tests (HajiRassouliha et al., 2018). DEAs require a high electric field to actuate (up to 120 V/μm). Although the CIDs are designed to shield the cells from the electric field, it is essential to separate the cell’s response to the mechanical stimulation from a possible unwanted electrical stimulation. In two previous studies, we investigated the influence of the electric field on cells (Poulin et al., 2016, 2018a), and found no observable impact due to the electric field. However, the cells in these previous studies were not human brain cells and we wanted to ensure the electric field also had no impact on the primary patient-matched cells we used for this study. Upon some testing we observed that our human brain cells did behave differently to the cells from our previous studies (Poulin et al., 2016, 2018a) (human lymphatic endothelial cells and rat ventricular cardiomyocytes). Hence, some of the CIDs were used as experimental control by attaching a circular adhesive to the bottom side of the membrane, thus immobilizing the membrane and stopping the CID from actuating when voltage is supplied. This allowed us to expose the control CIDs to the same electrical field than the stretched CIDs and to account for the potential effects of the electric field when assessing the change in gene regulation post-injury. Once we were able to normalize for any electric field effects, we conducted experiments to collect > 400 ng of RNA from each of the two time points (4 and 48 h) per condition (control and stretched) for qRT-PCR analysis.

### Quantitative real-time polymerase chain reaction protocol

RNA is collected at 4 and 48 h by lysing the cells using the Promega Lysis Buffer (Promega, WI, USA). The lysates were stored at −80°C until RNA purification was performed for qRT-PCR.

**FIGURE 4:**
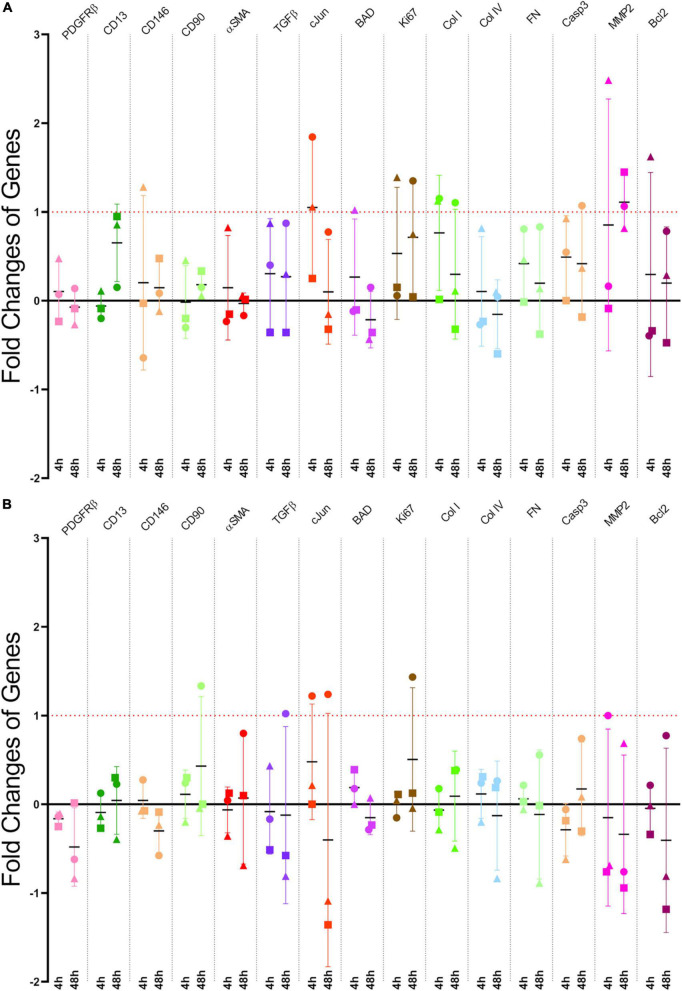
**(A)** Scratch assay gene fold changes: qRT-PCR Results from Pericyte Scratch Assays. Each point representing a different patient-derived cell line. **(B)** Stretching gene fold changes: qRT-PCR Results from Stretching Pericytes 50x with CIDs. Like the scratch assays, each point represents an individual cell line. That is, both models had *n* = 3. The horizontal black lines are the means, and the error bars are the standard deviations.

RNA purification was performed using the Ambion RNAqueous Micro Kit following the manufacturer’s instructions. RNA was treated with DNase from the ReliaPrep RNA cell Miniprep System Kit (Promega, WI, USA), and cDNA synthesis is performed using the Superscript^®^ III First-Strand Synthesis kit (Invitrogen, CA, USA). qRT-PCR was performed using Platinum^®^ SYBR^®^ Green qPCR SuperMix-UDG with Rox kit (Invitrogen, CA, USA), and analyzed according to the ΔC_*T*_ method (Livak and Schmittgen, 2001). The q-RT-PCR reactions were conducted in 386-well PCR plates (Invitrogen) using the QuantStudio 12K Flex system (Thermo Fisher Scientific) and comprised of the initial denaturation step, followed by 40 amplification cycles, and a final temperature ramp phase for the melt curve analysis, where we quality-checked for a single amplicon for each primer reaction. The level of gene expression is normalized to a housekeeping gene glyceraldehyde-3-phosphate dehydrogenase (GAPDH) at the 4 and 48 h time points. Any gene expression changes that exceeded a twofold change (i.e., > 1 CT) was deemed biologically meaningful. The primers used in the analysis is listed in Supplementary Table 1 and presented in the proceeding results section (Figure 4).

## Results and discussion

### Impact of design parameter optimization

**FIGURE 5:**
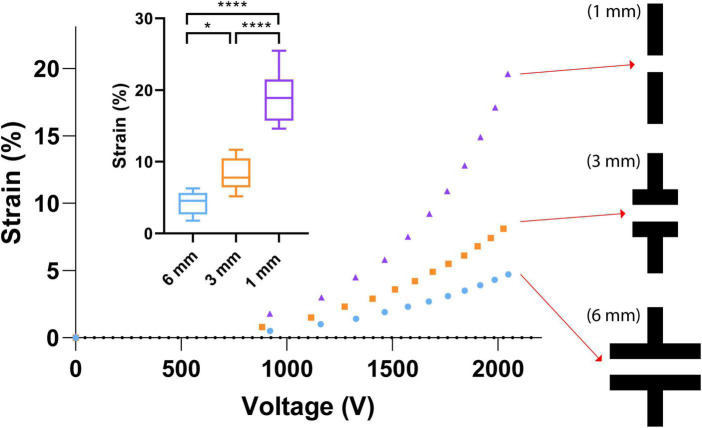
Electrode geometry effects. Small graph—the strain capabilities of each electrode design with an applied voltage of 2,000 V. A total of 45 CIDs were tested to produce the illustrated boxplots that display the effect of the electrode design on strain. The stars and quantity illustrate statistical significance and level of significant difference, respectively. The Brown Forsythe and Welch ANOVA test was used as there were more than two group samples and there was heterogeneity of variance between these groups (*P = 0.038, ****P < 0.001). Large graph—Three different electrode designs at the center of our CIDs were investigated in this study. The three designs are 6, 3, and 1 mm in width, which consistently produce 4, 8, and 20% strains, respectively, with an applied voltage of 2,000 V. This was a safe voltage that enables the device to survive the stretching tests without dielectric breakdown. In air, these devices can be function at much higher voltages, but the presence of cell culture media on the topside of the membrane limits the voltage range during testing.

Three different electrode designs were tested (6, 3, and 1 mm width), which consistently produce 4, 8, and 20% strains, respectively (Figure 5) with an applied voltage of 2,000 V. This was tested a safe voltage enabling the device to survive the stretching test without dielectric breakdown. In total, 45 CIDs were tested to illustrate the effect of the electrode design on strain. In air, the CID can be driven at much higher voltages, but the presence of cell culture media on the topside of the membrane limits the voltage range during the test, as DEAs in a humid environment are prone to dielectric breakdown at a much lower electric field (Albuquerque and Shea, 2020).

The final design we chose to implement is the CID with the 1 mm wide electrode (Figure 5), which generated the desired strain level (20%). One trade-off, however, was the decreased size of the active zone, which reduced the total amount of cells that can be injured on each CID due to the reduction of the size of active zones. This limitation was overcome by mass-fabricating more CIDs to ensure sufficient amount of RNA for our gene expression analysis via PCR. Currently, based on the cell culturing protocol described in the cell culture protocol section, each CID cultivates approximately 26,250 pericyte cells (plating 35 μL at a cell density of 750,000 cells/mL), which results in approximately 450 ng of available RNA for qRT-PCR use.

**FIGURE 6:**
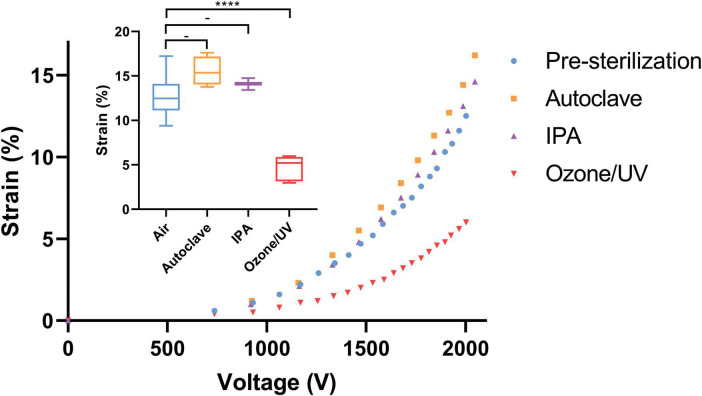
Effects of sterilization methods on CID strain. Small graph—summary of the three sterilization methods and its effects on strain relative to pre-sterilization at 2,000 V. Autoclaving and IPA had no detrimental effects on strain, whereas Ozone/UV treatment did and resulted in approximately half the initial strain capability. The stars and quantity illustrate statistical significance and level of significant difference, respectively. The dash indicates no statistical significance between the respective groups. The Brown Forsythe and Welch ANOVA test was used as there were more than two group samples and there was heterogeneity of variance between these groups (-*P* = 0.076 and 0.17 for pre-sterilization vs. autoclave and pre-sterilization vs. IPA, respectively. ****P < 0.001). Large graph—representative characterization curves of strain vs. voltage for each sterilization method. Again, showing how autoclaving and IPA had no adverse effects on strain, unlike Ozone/UV treatment that could only reach half of the initial strain capability pre-sterilization i.e., control.

Among the three sterilization methods tested—Ozone/UV, IPA, and autoclaving—we compared the strain capabilities of the CIDs post and pre-sterilization. The results show that Ozone/UV treatment is harmful and considerably deteriorated the strain capacity of the CIDs, while IPA and autoclaving had negligible effects when its strain capabilities were measured in air post-sterilization and pre-incubation (Figure 6). The degrading effects of Ozone/UV treatment on the actuation of our devices were observable, where at 2,000 V the device is only reaching approximately 5% strain (Figure 6). Whereas the devices sterilized with IPA or autoclaving had no degradation on their actuation capabilities, with both methods still reaching approximately 15% strain at 2,000 V (Figure 6). These optimization procedures provide confidence and consistency in our experiments, having established the sterilization process that does not deteriorate the strain capability of our CIDs. Between IPA and autoclaving, we chose autoclaving as the optimal sterilization method for practicality. Although IPA treatment does not have detrimental effects as Ozone/UV, it is still less practical because it involves a full immersion of the CIDs. Despite the adequate sterilization, the IPA treatment increases risk of short-circuiting the device if not fully dried properly.

One of the major disadvantages of using the autoclaving method compared to Ozone/UV or IPA treatment is that it requires more preparation time. This was overcome by streamlining the whole fabrication procedure.

### Stretch injury model

There is a paucity of information on the role that human pericyte plays in traumatic brain injuries in the literature, especially in terms of gene expression information. In order to address this gap, we utilized a well-established cell injury model, scratch assays, to identify and validate the target genes of interest involved in injury response.

We conducted scratch assays in the manner described previously in the scratch assay protocol section (Figure 1E and Supplementary Figure 2). Three tests, each test being a different patient cell line, were conducted for both the scratch assays and stretch injury model, which resulted in a total of six tests. These experiments allowed multiple genes to be examined simultaneously via PCR technology. The genes that were investigated included pericyte, inflammatory, fibrotic, and apoptotic markers. The selected genes were examined in the same manner as previously described in the qRT-PCR section. The PCR results from our scratch assays (Figure 4A) indicate that one of the most upregulated gene due to scratch injury is c-Jun, a transcription factor that modulates cellular stress signals and cell death (Wisdom et al., 1999; Pearson et al., 2003; Raivich, 2008), with over a twofold increase in gene expression when analyzed using the ΔC_*T*_ method (Livak and Schmittgen, 2001).

Although the traumatic lacerations experienced by the pericytes in these scratch assays are different from the stretch assay in our CID injury model, especially in the injury severity, they are both results of mechanical insults to pericytes. Moreover, the widespread use and the ease of conducting scratch assays provided relatively fast screening of numerous genes. Subsequently, this result gives us confidence in the genes that we examined when we conducted our CID injury model.

**FIGURE 7:**
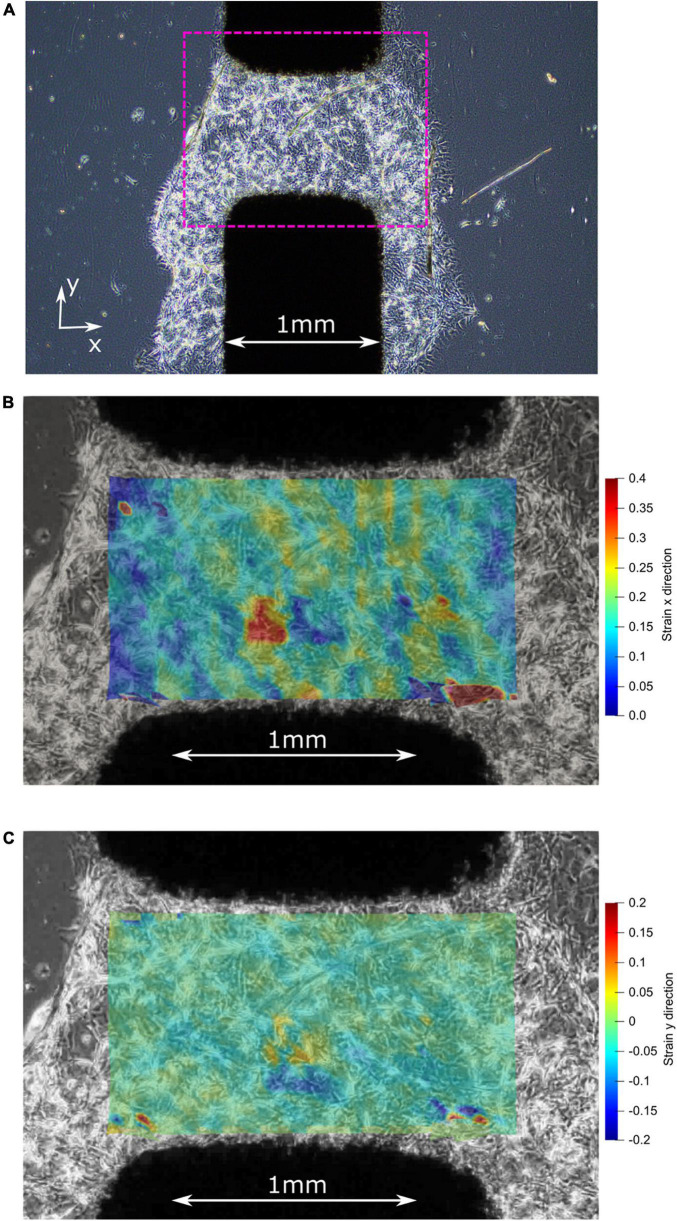
**(A)** Before stretching: center of the active zone on the CID with plated 3D matrigel and pericytes. **(B)** After stretching: strain map illustrating the high strain (up to 40%) experienced by the pericyte cells in the x-direction. **(C)** After stretching: strain map illustrating the low strain (mostly 0%) experienced by the pericyte cells in the y-direction, which is the direction of the initial pre-stretch during CID fabrication.

One of the most important factors of *in vitro* CIDs is the ability to apply homogenous strain to the cell seeded substrate. We used the video footages captured (c.f. Supplementary Video File) during the experiment to produce a strain map to analyze the strain field developed in the active area (Figure 7). Additionally, we acknowledge there are the occasional cell growth beyond the active zone of the 1 mm wide electrode, which could potentially affect the results. However, given the majority of cells plated are within the active zone, our evaluation has found this effect to be negligible.

The adherence of the pericytes to the silicone membrane with the assistance of Matrigel^®^, before stretching, is illustrated by the first image in Figure 7A where the Matrigel and pericyte mixture formed a 3D cell culture around the active zone of the CID. Once stretched, the pericytes within the active zone of the membrane experience at least by 20% strain (Figure 7B), while some cells were reaching strains up to 40% strain, as shown in Figure 7B, which is favorable as the study’s objective was to induce strains of at least 20%. The stretch produced by the CID is essentially uniaxial because of the minimal strain occurring in the y-direction, as illustrated in the figure’s third and final image (Figure 7C). Furthermore, the orientation of the cells relative to the uniaxial strain is non-specific, which reflects *in vivo* conditions. Thus, giving our injury model a higher fidelity to real-life mTBI than if we were to orientate all the pericytes in the same orientation.

We demonstrate that the strain induced resulted in upregulation in gene expression at both the 4 and 48 h-time points post-stretch, especially the *JUN gene* which express the transcription factor, c-Jun. This transcription factor modulates stress signals and is part of the c-Jun N-terminal kinase (JNK) pathway, which can lead to cellular death via apoptosis (Dragunow et al., 1993; Kyriakis et al., 1994; Wisdom et al., 1999). The upregulation of *JUN* expression implies that that patient cell lines are responding to the mechanical stretching of our CID (Figure 4B). *JUN* expression was also changed in our scratch assays (Figure 4A), but unlike in the more severe injury model of a scratch assay, where all cell lines upregulated *JUN* expression, the strain-induced through our CID, which represents a milder form of TBI, resulted in varied responses from different patient samples. One patient case resulted in a substantial upregulation of *JUN*, while two patient samples showed a substantial down-regulation at the 48 h time point (Figure 4B). This observation matches well with the clinical sequalae of mTBI which are manifested in a highly heterogenous manner (Cassidy et al., 2004; Lingsma et al., 2010; Hiploylee et al., 2017; Kenzie et al., 2017). Indeed, this highlights the benefits of working with primary cell lines that better recapitulate the actual patient population. Nevertheless, in the context of TBI, the effects of modulating the JNK pathway have been done on human tissue by Ortolano et al. (2009), and similarly with *in vivo* animal models by Yatsushige et al. (2007) and Rehman et al. (2018). The results of these studies suggest that JNK pathway inhibition may be a potential therapeutic target for TBI (Yatsushige et al., 2007; Ortolano et al., 2009; Rehman et al., 2018). Although much more work needs to be done, patient cells that are down-regulating *JUN* may have an innate ability to enable cytoprotective mechanisms in response to milder injury, whilst the case that up-regulated *JUN* may be more prone to TBI-induced injury and require earlier therapeutic intervention. Further repeats and a deeper analysis between patient cell lines are currently underway to clarify these differences in various patient-derived cell lines.

In contrast to c-Jun, the other apoptotic markers namely B-cell lymphoma 2 (Bcl-2), Bcl-2 associated agonist of cell death (BAD), transforming growth factor-beta (TGFβ), and caspase 3 (C3) did not respond to the mechanical stretching with substantial upregulation (Figure 4B). On the contrary, a recent study by Deng et al. (2020) published clinical data that suggests that Bcl-2 significantly upregulates in TBI patients, as well as Bcl-2 and C3 having on average greater responses in our scratch assays (Figure 4A). Moreover, Rosas-Hernandez et al. (2018) found that with 10 and 15% high speed stretch, they were able to induce apoptosis via the activation of caspase 3, a protein that acts as a mediator of programmed cell death. Whereas we did not see significant upregulation of caspase 3 in our stretch model. However, Rosas-Hernandez et al. (2018) used rat brain microvascular endothelial cells compared to our primary human brain pericytes, which could explain these potential differences. Another difference in injury model is the number of repeats implemented, where we repeated our mechanical stretch 50x compared to their single stretch.

Several of the analyzed genes are classical pericyte markers, namely, PDGFRβ, CD13, CD146, and αSMA (Smyth et al., 2018b). These genes did not change significantly at either time point in response to the mechanical stretching (Figure 4B), which corresponded with our scratch assay results (Figure 4A).

Similarly, the scarring/fibrotic markers of type I collagen (Col I), type IV collagen (Col IV), fibronectin (FN), and matrix metalloproteinase-2 (MMP-2), none responded substantially to mechanical stretching bar one case at 4 h (Figure 4B). This lack of response was in contrast to previous studies that showed pericytes are involved in the scarring process in response to injury (Goritz et al., 2011; Greenhalgh et al., 2013; Soderblom et al., 2013; Trost et al., 2016; Dias and Göritz, 2018).

Furthermore, because our scratch assay also showed greater responses for Col I and FN (Figure 4A), this might indicate that these genes are activated in a magnitude-dependent manner after a certain threshold beyond the 20% that we applied. Another possibility is that the time points in our study (4 and 48 h) might not have enough resolution to capture the delayed responses in a stretch injury model. A future study will investigate multiple time points and strain magnitudes to fully characterize activation patterns of these genes.

Likewise, Ki-67, a protein that is a cellular marker for proliferation also did not respond significantly to stretching from our injury model, with the exception of one case at 48 h (Figure 4B). Whereas our scratch assay did have a meaningful upregulation at both 4 and 48 h, which may imply that injuries that lead to the creation of lesion encourages proliferation more than a milder injury of stretching for pericytes (Goritz et al., 2011).

Moreover, due to the paucity of literature of stretch injury models that utilized patient-matched human brain pericytes, it is difficult to compare directly with previous studies. However, our model holds great promise as it is functional in both an engineering and biological context. Therefore, providing a platform that has been characterized and ready to be utilized for future studies.

The future work would include further development into a multi-well system. Fabricating a multi-well CID should alleviate some of the current cell culturing protocol’s limitations, and overall improve our injury model. With a multi-well system, throughput would increase via greater cell culture capacity where the quantity of cells, and consequently RNA, would increase per CID. This throughput increase would also be achieved without compromising on the device’s ability of reaching a high strain threshold. We are in the process of developing a multi-well system which will be reported in our subsequent publication.

Another limitation of our current design is the use of simple square signals in applying strains to cells. However, DEAs are capable of recreating physiological strain profiles (Poulin et al., 2018a), and the square signals used in this current study was for validation purposes. Moreover, previous studies have shown that the duration of head impact as well as the magnitude play an important role in triggering secondary responses of TBI (Tagge et al., 2018), indicating that complex strain profiles are required to recapitulate *in vivo* like impact situations. Our future work will include the incorporation of such complex strain profiles in our CID device. Additionally, we acknowledge that mTBI involves not only pericytes but also other cells of the NVU. Therefore, co-culturing with other NVU cell types like astrocytes or microglia will be the aim of our future studies. Thus, increasing the fidelity of this injury model to better capture the complexity of mTBI. Furthermore, the slight growth of the cells outside of the active zone may have potentially been another cause for the heterogeneous responses of our experiments, in addition to the variability of patient-specific responses. This optimization issue is being addressed and will be implemented in future studies to ensure a more consistent and robust injury model. Similarly, the marginal heterogeneity of the cells (Supplementary Figures 2, 3) may have also contributed to the difference in gene expression responses, in addition to the variability of patient-specific responses. It is worth noting, αSMA do not necessarily have to co-localize with PDGFRβ in capillary pericytes as demonstrated in our previous work (Smyth et al., 2018b).

## Conclusion

We have demonstrated the first *in vitro* mTBI stretch model based on human brain pericytes. Collectively, our results demonstrate that the CID that we developed is capable of simulating milder forms of injury in an *in vitro* stretch model. Our CID device can produce 20% mechanical strains and apply repetitive mechanical insults to cells for an extended period of time. These parameters can induce biologically meaningful changes in primary human brain pericytes that reflect patient variability. This method of studying mTBI shows excellent promise for elucidating minute but important biological changes that may lead to mTBI-induced complications. Such a device would be a valuable tool for the identification of therapeutic targets, or for the establishment of a damage strain threshold, once we apply realistic TBI strain profiles.

## Data availability statement

The original contributions presented in this study are included in the article/Supplementary material, further inquiries can be directed to the corresponding author/s.

## Author contributions

Y-HW, TP, MD, VS, and SR designed the study and analyzed the data. Y-HW performed the experiment and wrote the first draft of the manuscript. TP, MD, VS, and SR reviewed and edited the manuscript. PS consented patients, provided surgical tissue and patient information, and reviewed the manuscript. EK designed the scratch assay. SF contribution to assisted with cell culture preparation. VS and MD provided funding. All authors contributed to the article and approved the submitted version.
