# Supplementary material for: Analyzing pericytes under mild traumatic brain injury using 3D cultures and dielectric elastomer actuators
Source: Front Neurosci. 2022 Nov 10;16:994251. doi: 10.3389/fnins.2022.994251 (PMC9684674; doi:10.3389/fnins.2022.994251)
Supplement: Supplementary file 1 [file Data_Sheet_1.docx]

# Supplementary Figures

(a)


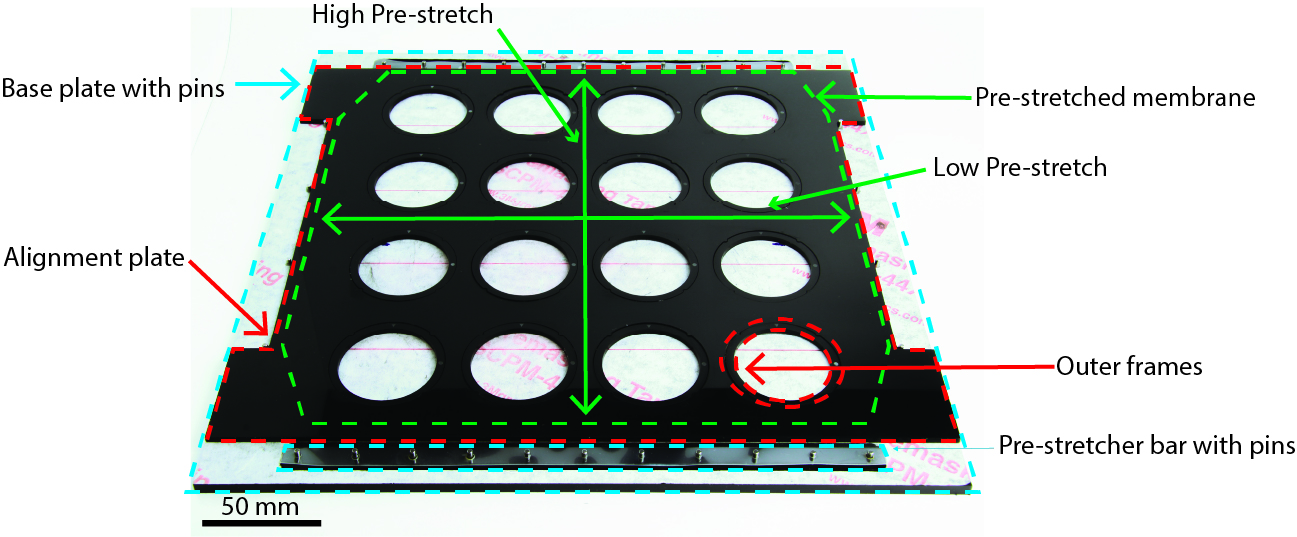


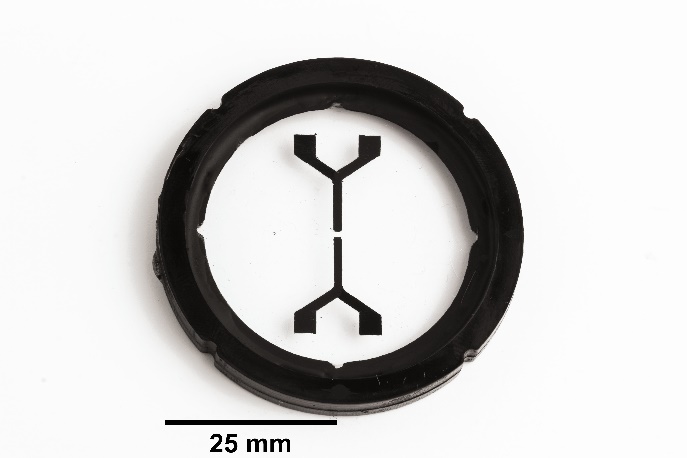

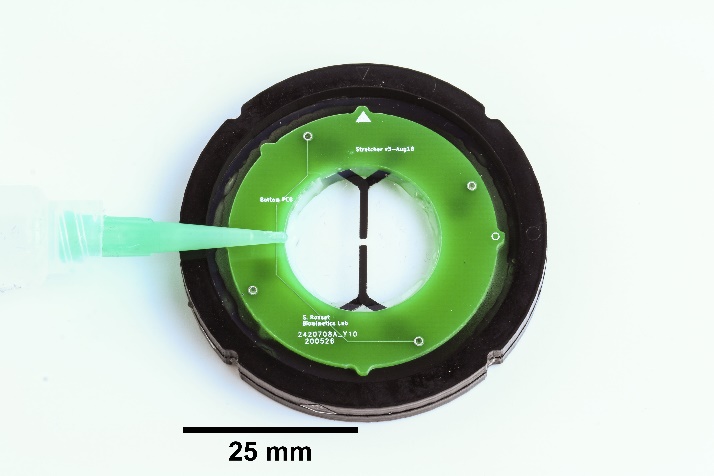

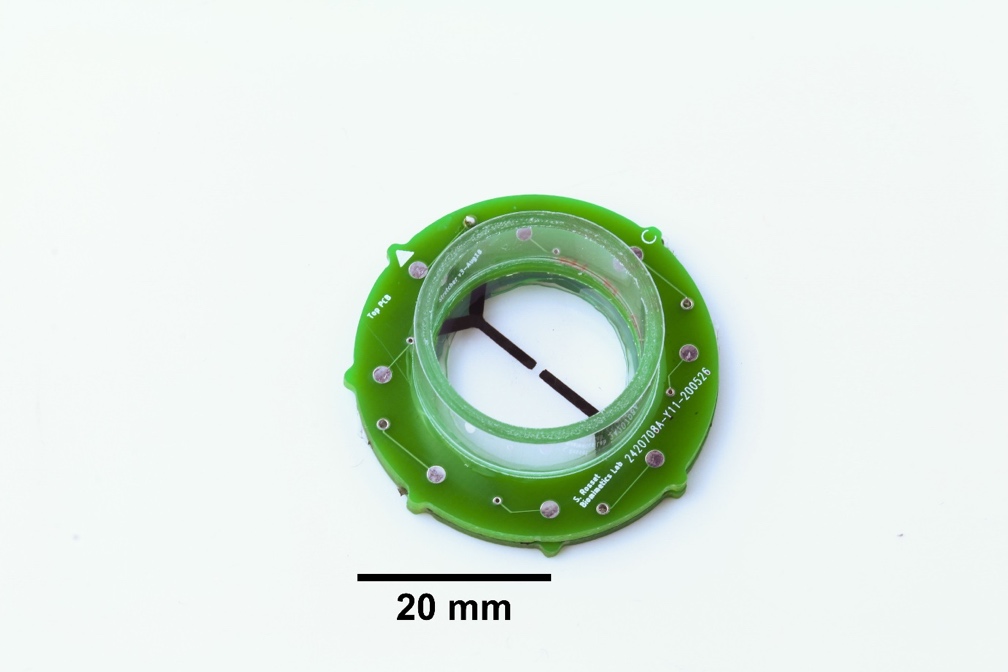


(d)

(c)

(b)

Figure S1 (a) Wacker Elastosil® film 2030 silicone membrane is biaxially pre-stretched 2.7 and 1.2 times its original lengths along the high and low pre-stretch directions, respectively. The 16 hole alignment plate is then placed over the membrane to guide the attachment of each 52mm-diameter outer frames. (b) The compliant electrodes are made via spray-coating the membrane with an electrically conductive ink through a shadow mask, and is repeated for both sides of the membrane before being cured at 80°C for 30 min. (c) Printed circuit boards are glued to both sides of the membrane and provides a structural frame for holding the membrane pre-stretch, as well as the electrical connection to the compliant electrodes. The bottom side of the PCBs are in contact with the membrane and have large square metallic contact pads that align with the carbon electrodes. Application of a conductive silicone paste (Silicone Solutions® SS-24 Electrically Conductive Silicone RTV) on the contact pads prior to gluing PCBs to the membrane ensures good electrical contact. (d) The membrane between the outer diameter of the PCB and outer frame is cut before this initial frame is discarded.


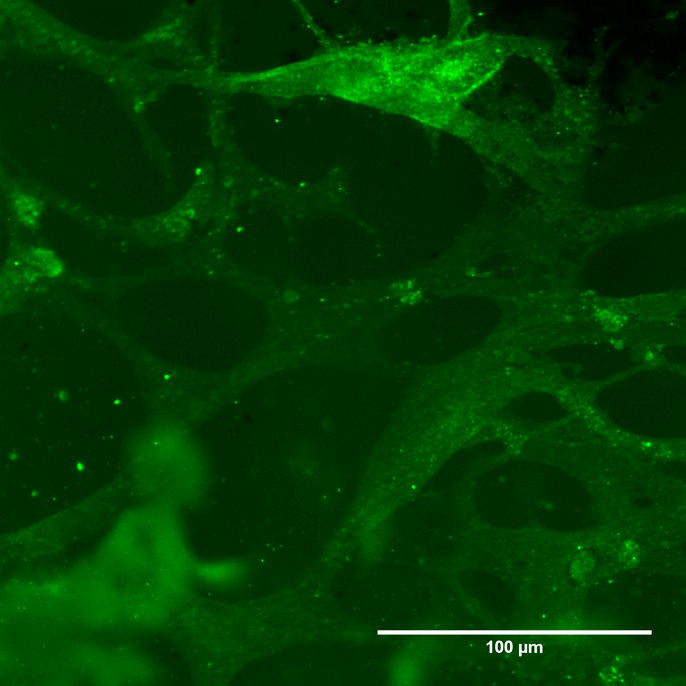

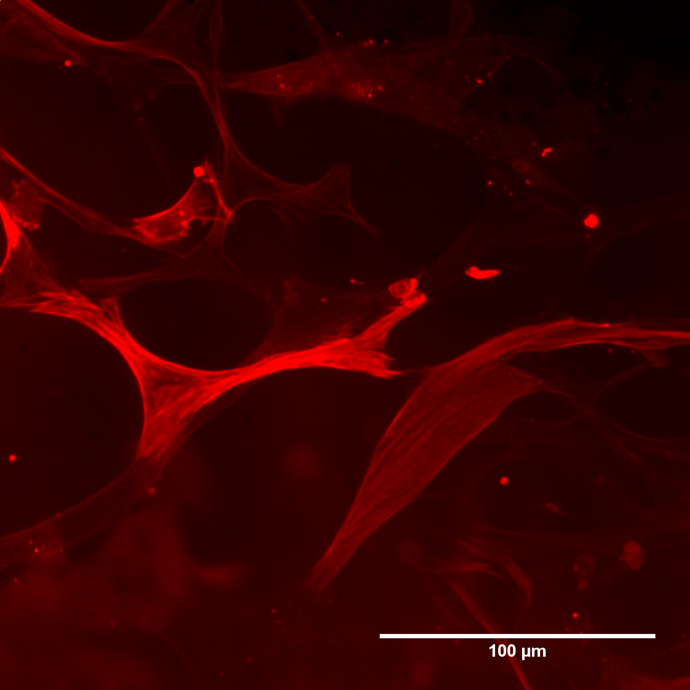


(c)

(a)

(b)


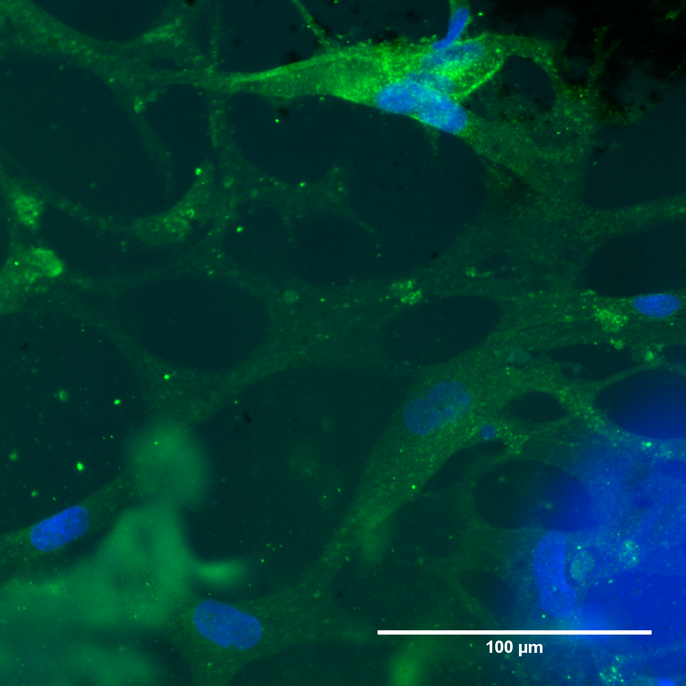


Figure S2 (a) PDGFRβ immunostaining of experimental patient-derived brain pericytes (b) αSMA immunostaining of experimental patient-derived brain pericytes (c) PDGFRβ and associated Hoechst positive nuclei immunostaining


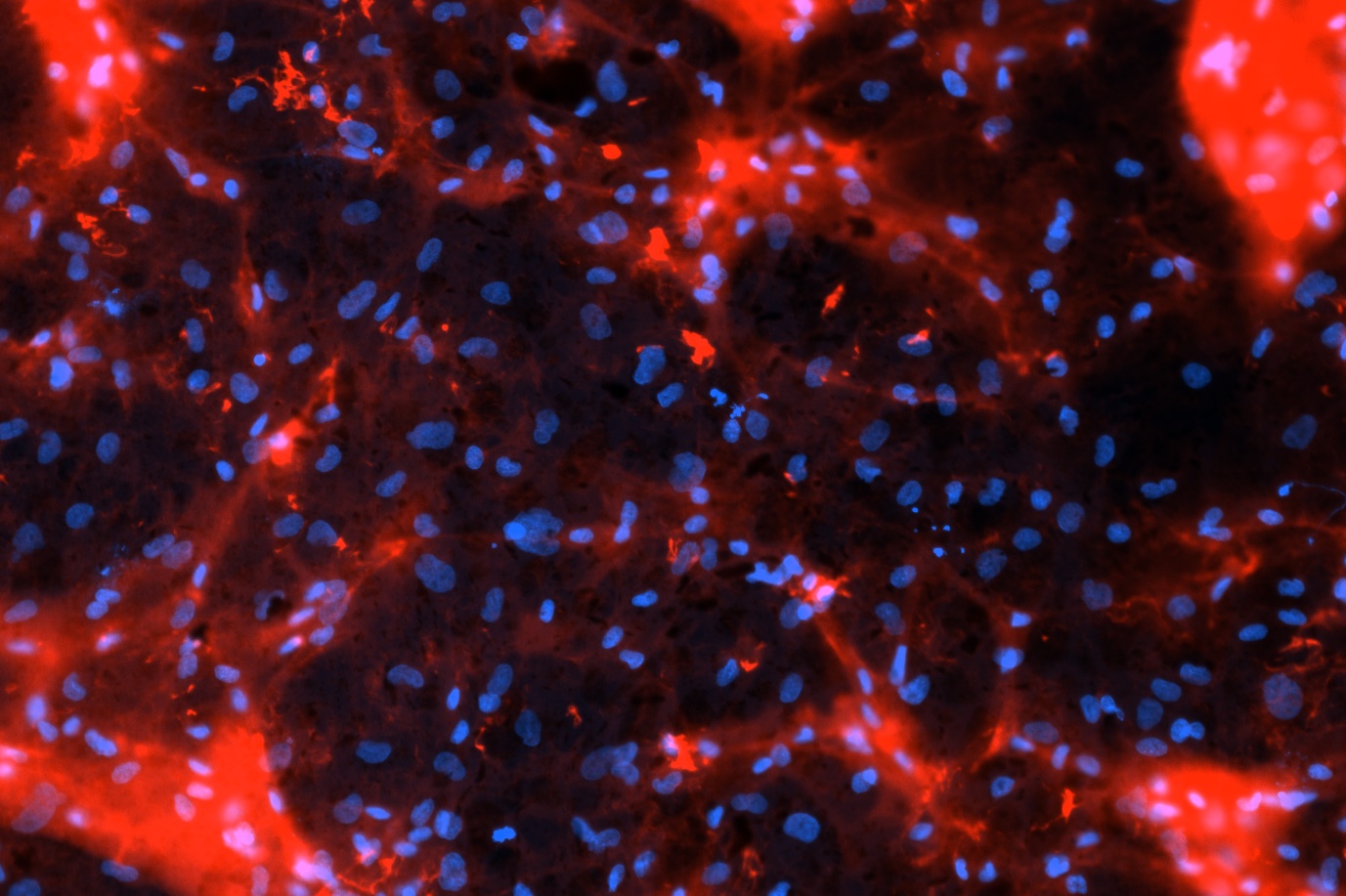


(a)

(b)


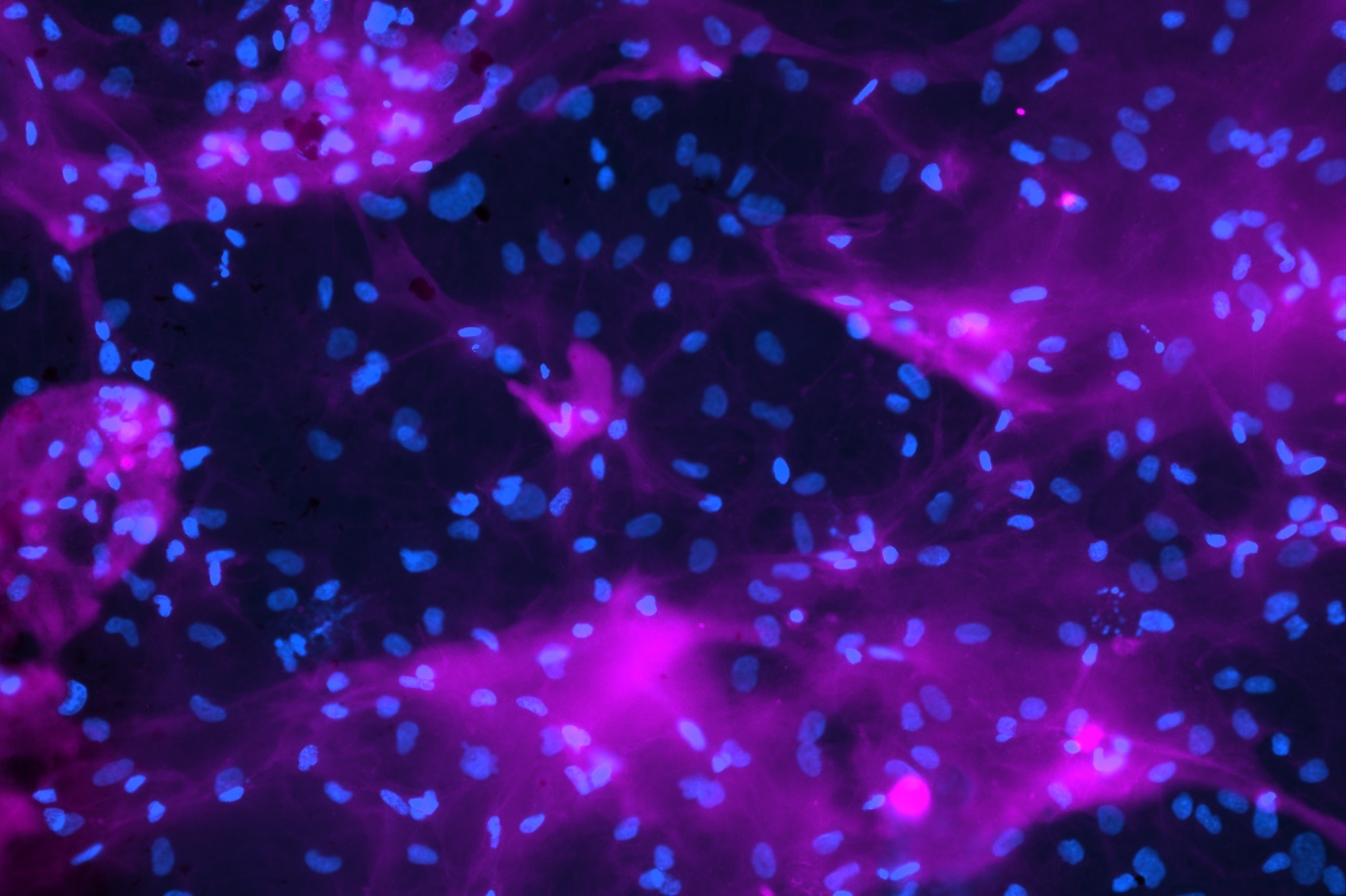


Figure S3 Even distribution of pericytes on the CID (scale bar = 100 um). (a) αSMA immunostaining (a) PDGFRβ immunostaining


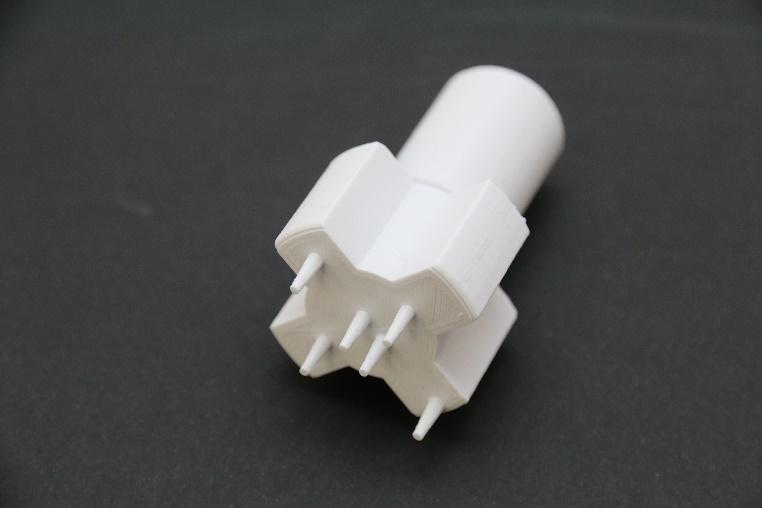


Figure S4 Custom-made scratch device with a handle and six pins that fit snuggly into a well on a 6-well plate. Designed to inflict six circular lacerations on the pericyte monolayer when rotated by the user.

Table S1 List of primers used for qRT-PCR in scratch assays and stretch injury model

Primers List used

| **Gene** | **Protein** | **Sequence (5’ to 3’)** | | **Amplicon (bp)** |
| --- | --- | --- | --- | --- |
| *GAPDH* | GAPDH | Fw | CATGAGAAGTATGACAACAGCCT | 113 |
|  |  | Rv | AGTCCTTCCACGATACCAAAGT |  |
| *PDGFRβ* | PDGFRβ | Fw | CGCAAAGAAAGTGGGCGGCT | 80 |
|  |  | Rv | TGCAGGATGGAGCGGATGTGGT |  |
| *CD13* | CD13 | Fw | ACCTGGGTGCTGACTATGCGGA | 82 |
|  |  | Rv | ACTGCCATCACGCGGTACACA |  |
| *CD146* | CD146 | Fw | AGTCCCAAGGCAACCTCAGCCA | 76 |
|  |  | Rv | CGCACACGGAAGATGAGCGT |  |
| *CD90* | CD90 | Fw | AGCAAGGACGAGGGCACCTACA | 68 |
|  |  | Rv | TGGGAGGAGATGGGTGGGGAAT |  |
| *ACTA2* | αSMA | Fw | ACGTGGGTGACGAAGCACAGA | 84 |
|  |  | Rv | CGTCCCAGTTGGTGATGATGCC |  |
| *TGFβ* | TGFβ | Fw | GGTCCTGGCCCTTTACAACA | 90 |
|  |  | Rv | GACTCGGTGTTTTCCTGGGT |  |
| *CJUN* | c-Jun | Fw | ATCCAGTCCAGCAACGGGCA | 89 |
|  |  | Rv | TGCGGTTCCTCATGCGCTTC |  |
| *BAD* | BAD | Fw | CGGAGGATGAGTGACGAGTT | 135 |
|  |  | Rv | CAAGTTCCGATCCCACCAGG |  |
| *KI67* | Ki67 | Fw | AGCAAGCGGTGATGAGGGCA | 79 |
|  |  | Rv | TGCTGGAGCCGGTGTCTGTT |  |
| *COLI* | Col I | Fw | TGGATGAGGACCAGAAAGTTCGGC | 80 |
|  |  | Rv | ACTGAGCATCACCCTGGACGTGT |  |
| *COLIV* | Col IV | Fw | CCCGAAAGGCCAGCAAGGTGTT | 77 |
|  |  | Rv | GGGCACCGTCAAACCCAGGAAT |  |
| *FN1* | Fibronectin | Fw | CGAGAGTGCCCCTACTACAC | 84 |
|  |  | Rv | TGTTGGTGAATCGCAGGTCA |  |
| *CASPASE3* | Caspase3 | Fw | GGTGCTATTGTGAGGCGGTT | 74 |
|  |  | Rv | CCACGGATACACAGCCACAG |  |
| *MMP2* | MMP-2 | Fw | GCAGTGGGGGCTTAAGAAGA | 77 |
|  |  | Rv | AGCTGGTTGGTTCATGCACT |  |
| *BCL2* | Bcl2 | Fw | ACTGGGGGAGGATTGTGGCCTT | 70 |
|  |  | Rv | ATCTCCCGGTTGACGCTCTCCA |  |
